# How Are We Facing It? Dispatches From Pathology Residents in a COVID-19 Lombardy Hospital

**DOI:** 10.3389/fpubh.2020.00259

**Published:** 2020-06-04

**Authors:** Miriam Cieri, Camilla De Carlo, Marina Valeri, Vincenzo Belsito, Cesare Lancellotti, Massimo Roncalli, Piergiuseppe Colombo

**Affiliations:** ^1^Department of Pathology, Humanitas Clinical and Research Center IRCCS, Milan, Italy; ^2^Department of Biomedical Sciences, Humanitas University, Milan, Italy

**Keywords:** resident, COVID-19, pathology, healthcare worker, autopsy, smart work, learning, safety

## Abstract

At the end of February, the Italian National Health Service reported a hot spot of Coronavirus disease in the Lombardy region. COVID-19 is a highly pathogenic viral infection which poses some challenges for healthcare workers. Indeed, Pathology Departments are involved in reorganizing samples' management, from their delivery until their processing, according to National and WHO guidelines. Since Lombardy has been declared COVID-19 hot spot, due to decreasing number of surgical procedures, our Department adopted a policy to reduce personnel, allowing pathologists to work remotely during the outbreak. Lacking clear information about viral load on tissue samples, all human specimens must be considered potentially infectious, as well as patients during post-mortem examinations, and clinical information on COVID-19 status is mandatory. It is also important that Pathology staff receive an adequate training, and adherence to rules should be always accompanied by common sense.

## Introduction

After the first reported case in December 2019 in Wuhan (Hubei Province, China), SARS-CoV2 is now considered one of the major pathogens that primarily targets the respiratory system and in a few months has become a global health concern for humans. The virus spreads via droplets, hands and remains on unanimated surfaces at room temperature for up to 9 days. Incubation time is between 2 and 14 days (1, 2).

This is the reason why this novel coronavirus poses an occupational health risk to healthcare workers. In this regard, our country paid a heavy price with more than 200,000 infections among general population, of which 17,000 in healthcare workers and 200 deaths, including general practitioners, hospital physicians, nurses and social assistants (3).

Hence, the COVID-19 outbreak made it necessary for all healthcare providers to reshape their daily routine, in order to keep safe distances, limit worker exposure to infectious risk and maintain ordinary activities. To achieve this goal, different aspects of our daily life changed drastically.

As Italian residents of a Surgical Pathology Department in a heavily impacted region by COVID-19 infection, we feel the need to share our experience in this new setting. To this aim we illustrate major changes and safety measures undertaken in our hospital and specifically in our Department.

In this brief paper we will discuss general safety measures, changes in grossing and post-mortem procedures and new planning of daily work in the outbreak.

## General Measures Adopted in Our Hospital

The rapid spread of the virus raised concerns over the outbreak modalities in Italy and led to the urgency to re-modulate healthcare units. Extraordinary measures have been adopted to minimize risk of infection among patients and healthcare workers: the hospital cafeteria was closed; the capacity of the canteen has been reduced to increase interpersonal distances (>1 meter); several check-point areas were established in order to provide surgical masks and alcoholic solutions for hand sanitizing, and to check temperature at the entrance.

In this scenario, a decrease in the number of planned surgery interventions was programmed by the hospital direction, limiting the activity to not-deferrable ones, such as neurovascular and oncologic procedures. Notably, all the candidates for surgical procedures were restricted to asymptomatics and preoperatively screened by lung CT.

The number of cases decreased from 4,500 to 1,500 specimens/month, and cytological cases decreased from 2,500 to 500 specimens/month. Nevertheless, during the entire pandemic period, our Department has received and is still receiving on a daily basis a significant number of routine formalin fixed specimens from surgery. Fresh samples for intraoperative examination were sent to the Department to ensure medical care for oncologic patients requiring urgent treatments.

## Gross Room, Frozen Section Examination and Tissue Biobank

Following the Covid-19 pandemic outbreak, residents, and Pathologists have taken special care and precautions in every medical space to prevent the spread of coronavirus and to make gross sampling and frozen section procedure as safe as possible.

One of the first rules was to restrict the number of personnel inside the laboratory to the utmost necessary and keeping a safety distance among operators. All the doors had to be closed. Senior consultants and medical students were asked not to attend the lab; technicians and medical staff planned their daily routine based on the maximum number of individuals determined for each room.

**Figure 1:**
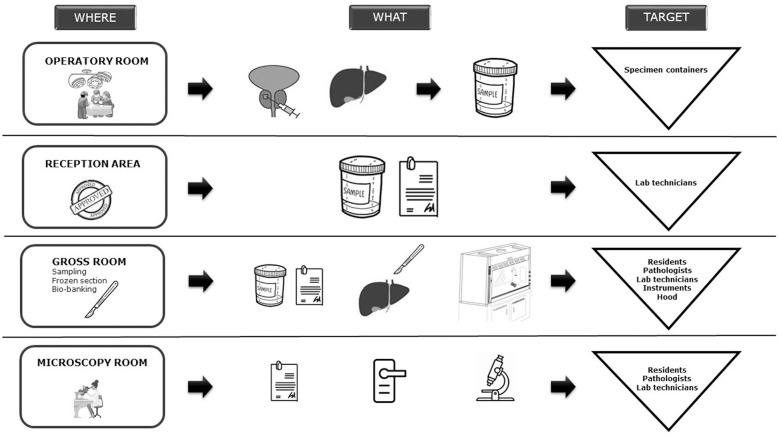
Chain of contagion in a Pathology Department.

Procedures operated in the Department that had to be considered at high risk of contagion were: delivery of samples from operative rooms, manipulation of specimen in the gross room, frozen section examination, and bio-banking, as shown in Figure 1. Based on the recommendations from the Italian Pathology Society (SIAPEC), all specimens had to be considered potentially infectious and mobilized in “*ad hoc*” safety envelopes before entering the lab. In addition, all the samples, regardless of origin and pre-analytical manipulations, needed a detailed clinical information about COVID-19 infection in order to be accepted for processing (4). Prior sampling, special care was assured in fixing all the specimens in formalin for at least 24 h and for not <48 h if COVID-19 positive. Pathologists and assistant pathologists kept dressing surgical masks and protective glasses. Due to their limited availability, FFP2 masks and disposable lab coats were reserved to specific procedures requiring handling of fresh material (e.g., frozen sections, bio-banking procedures).

During the pandemic escalation, we mainly performed intraoperative frozen section examination on surgical margins during prostatectomy, radical cystectomy and pancreasectomy, for malignancy assessment of lesions, and sentinel lymph node status in breast cancer. In this case, FFP2 masks, and disposable lab coats were available to residents, staff pathologists, and technicians. For frozen section examination, we emphasize the importance of a dedicated cryostat which was kept closed during freezing and cutting to avoid vaporization. After the procedure, samples were immediately fixed in formalin and the instruments disinfected in 2–3% hypochlorite solution for 24 h.

In our Department, in an ordinary day, bio-banking is a time-consuming activity (about 2 h per day); all types of solid tumors are collected. However, in the pandemic emergency, according to our national guidelines, the procedure was not performed on suspected or positive COVID19 patients (4). In case of tissue bio-banking, FFP2 masks, surgical gloves, disposable lab coats, and protective glasses were made available to residents; at the end of the procedure, all the instruments and hoods surfaces were disinfected with hypochlorite solution.

## Autopsy Procedure

SIAPEC recommendations for post-mortem examinations of patients with suspected, probable or confirmed COVID-19 were published on March 2020, indicating that all subjects undergoing postmortem exams have to be considered potentially infectious so that a number of precautionary measures should be always in place (5). Specifically autopsies in biosafety 3 group (hazard group 3) should be performed by two operators—one of them acting as the “clean” assistant- and the other as technician, in Airborne Infection Isolation Rooms (AIIRs). AIIRs consist of rooms with negative pressure and at least 6–12 air exchanges per hour with air being expelled outside directly or through a HEPA filter.

Physicians and technicians must dress and undress in a designated filter zone, following the Center for Disease Control and Prevention (CDC) Sequence for Donning and Removing Personal Protective Equipment. Single-use cap, gown and overshoes, a double pair of disposable gloves, cut-resistant gloves, FFP2 or FFP3 masks and a facial protection must be used (5, 6). Our Pathology Society highly recommends limiting the involvement of residents in this activity. However, residents can contribute under senior pathologists' supervision but not in high-risk procedures, such as evisceration. When a dissection room is not properly equipped, the decedent is sent to one of the three reference centers in Lombardy (5). It was also recommended to perform an oropharyngeal swab within 2 h of death and with available results within 24 h, in patients deemed not to have SARS-CoV-2 infection. If this test is unavailable, the autopsy must not be performed. In case of positive test or when COVID-19 is clinically or radiologically confirmed, an interaction among pathologists and clinicians is highly recommended to decide whether or not to carry out the procedure. Since a negative pressure dissection room and a proper filter zone is not available in our Institution, we are not performing autopsy of SARS-Cov-2 positive patients. On the other hand, we are using biosafety 3 PPE for autopsies of patients with negative tests (5).

## New Challenges in the Daily Work of Residents in Pathology

Because the oncological elective surgery rapidly dropped to 70%, it was decided to take the opportunity to experience new models of work. Indeed, the Pathology department had first to re-organize the logistic of work, to keep the distance between operators and to support the hospital in the patients' management. Accordingly, three substantial innovations have been introduced for the medical staff: smart working, digital pathology and check-point activity.

Senior pathologists have been offered the technology to be able to work remotely and they have scheduled a twice-a-week smart working activity, contributing to general safety measures. As a consequence, digital pathology, previously used only for research purposes, was routinely introduced also for resident training in histopathology, cytology virtual classes, multidisciplinary tumor boards and inter-institutional meetings (e.g., Sarcopatologi of Milan). With the partial conversion of glass slides into high-resolution digital data, we are now becoming familiar with a technique alternative to the microscope, and begin to appreciate its strengths during the slides review.

Specifically, from March 1st to the end of April, each Resident reviewed about 25 surgical cases/week with senior pathologists (mostly breast, gastrointestinal, pulmonary and urologic tumor pathology), representing about half of the work load of the Department in this period.

Embracing a new technology is not always easy, but due to both our relatively poor experience with the microscope and our reliability on digital tools, we approached digital pathology with impartial enthusiasm.

In fact, it makes much easier to review digital slides on the monitor, taking annotations and pictures when needed. Moreover, sharing links, tables, and charts in teams chat is now simple and immediate.

In addition, since we were not involved in the first line against the emergency, we volunteered to support clinicians' work in the check-point activity. We performed three easy, but rigorous, tasks: to provide protective equipment to every individual entering the hospital (such as surgical masks), to distribute hand sanitizer and to check body temperature, in order to detect potentially contagious people (temperature over 37.3°C is considered a red flag), suggesting they go home and contact their general practitioner.

## Discussion

The COVID-19 outbreak has brought changes in many aspects of social and working daily life. Milan, Bergamo and Brescia rapidly became hotspot areas and we believe our field experience could be useful for the disease management to institutions around the world (2).

Lombardy authorities and the emergency task force decided to avoid unnecessary scheduled surgeries. In this perspective, administrative staff reshaped hospital spaces. As a COVID-19 Hub, about 75% (250 beds) of our patients are COVID-19 positive. Moreover, about 90% of surgical blocks are converted into COVID-19 intensive care wards.

As a Pathology Department, we have been involved in reorganizing samples' management, from their delivery until their processing, according to National and WHO guidelines. Nowadays, lacking clear information about viral load, we must consider every patient potentially infectious and clinical information on COVID-19 status is mandatory. In this critical situation, we are adopting PPE and reserving frozen section only to COVID-19 negative ones (Figure 1).

In the United States the College of American Pathologist (CAP) has urged the Centers for Medicare & Medicaid Services (CMS) to allow pathologists to work remotely during the COVID-19 outbreak, and asked Congress to support this request. In addition, the CAP stated that all laboratories should be given the discretion to determine what is best for their staff in managing this pandemic (7). Based on the experience of our Hospital, we agree with CAP requests, and we suggest to adopt this policy for personnel's safety.

Pathology departments are facing an important challenge dealing with autopsy procedure. Indeed, it is unlikely to have a dissection room with a biosafety 3 level in every institution. Hence, following SIAPEC guidelines, we suggest performing autopsy procedures only in designated and equipped institutions (5).

As Pathology residents we conclude that, despite the multiple issues in our institution caused by the pandemic crisis, we also had the opportunity to ensure department service respecting social distancing rules, facing a new organization and dealing with technology. In fact, technology used for smart working in order to increase social distancing, and the use of digital pathology, find a new, interesting role in our routine, in diagnostic activity with a fruitful exchange experience among residents and tutor, and in remote training (e.g., webinar). It is possible that these recommendations will be likely adopted in the near future as daily routine (Table 1). Taking our experience into consideration, the more we work in safe conditions, following good practice rules, the more we take part in the “curve flattening” process.

**Table 1:** Summary of recommendations based on experience during the pandemic at Humanitas.

**People management**	**Equipment**	**Teaching and training**	**Samples handling**	**Autopsy**
•Personnel number reduction to the utmost necessary; relocation (check point activity) •Work schedule reshaping •Personnel distancing •Remote working •Clear and efficient communications	•PPE: surgical masks, FFP2, surgical gloves, disposable lab coats and glasses •Waste-sparing PPE management •Hypochlorite solutions •Alcoholic solutions •Technology: scanners, laptops, pairing softwares	•Virtual meeting to review slides with tutors •Training on digital pathology •Multidisciplinar board meeting •Webinars for Residents	•Fresh material handling with PPE (FFP2, FFP3 masks) •COVID-19 status assessment •Dedicated cryostat •Samples fixed in formalin for at least 24 h (48 h for COVID-19 +) •Instruments disinfection	•COVID-19 status assessment (swab within 2 h) •COVID-19: performed with PPE (FFP2 and FFP3) •High-risk procedures restricted to Senior Pathologists •COVID+: performed only in referred Pathology Department

## Data Availability Statement

The raw data supporting the conclusions of this article will be made available by the authors, without undue reservation.

## Author Contributions

CD, MV, VB, and CL drafted the manuscript and contributed to the critical revision. MC and PC contributed to the study conception and critical and final revision. MR contributed to the final revision.

## Conflict of Interest

The authors declare that the research was conducted in the absence of any commercial or financial relationships that could be construed as a potential conflict of interest.
